# “*I…Tell Her Not to Take Medicines*”: Understanding Engagement in the Prevention of Mother to Child Transmission (PMTCT) Care Continuum through the Socio-Ecological Model

**DOI:** 10.3390/ijerph192013530

**Published:** 2022-10-19

**Authors:** Anjali Modi, Cristian Garcia-Alcaraz, Sangita Trivedi, J. K. Kosambiya, Kristen J. Wells

**Affiliations:** 1 Department of Community Medicine, Government Medical College, Surat 395001, India; 2 San Diego State University/University of California San Diego Joint Doctoral Program in Clinical Psychology, San Diego, CA 92182, USA; 3 Department of Psychology, San Diego State University, 6363 Alvarado Court, Suite 103, San Diego, CA 92120, USA; 4 Department of Pediatrics, Government Medical College, Surat 395001, India

**Keywords:** Human Immunodeficiency Virus/acquired immuno-deficiency syndrome (HIV/AIDS), prevention of parent/mother to child transmission (PPTCT/PMTCT), elimination of parent/mother to child transmission (EMTCT/EPTCT), health behaviour, male involvement, qualitative

## Abstract

With ten percent of the world’s children living with Human Immunodeficiency Virus (HIV/ AIDS) in India, achieving elimination of parent/mother to-child transmission (EPTCT/EMTCT) is far away. Timely initiation and optimal adherence to the prevention of parent/mother to child transmission (PPTCT/PMTCT) may reduce new paediatric HIV infections to zero. This qualitative study applies the Socio-ecological Model (SEM) to understand country, region and context-specific factors influencing mothers’ engagement in the PMTCT care continuum. Maximum variation sampling and saturation tenets determined the sample size. An in-depth interview guide based on SEM “a priori” and emerging themes captured narratives of the parental dyad. The translated and transcribed audio records were coded by direct content analysis method, both manually and with Atlas Ti software. The coding reports were discussed for consensus and final analysis. Male partner, peers, community health workers (CHWs), hope for healthy baby, knowledge about HIV and preventive services, free anti-retroviral therapy, transportation and the early infant diagnosis (EID) tool influenced PMTCT care continuum. Testing and referral policies of the private sector facilitated internalized or self-stigma. Future interventions should seek to develop pregnant women’s support system by engaging male partners, peers, and CHWs. Strategies addressing private sector and community awareness about freely available HIV prevention and care programs may enable optimal PMTCT utilization.

## 1. Introduction

Ten percent of the world’s children living with the Human Immunodeficiency Virus infection or acquired immune-deficiency syndrome (HIV/AIDS) reside in India, a country experiencing the third largest HIV epidemic in the world [1,2]. Between 2000 and 2015, India reduced the annual rate of new paediatric HIV infections from an estimated 0.24 lakh to 0.10 lakhs (1 million is 10 lakhs in Indian terminology) [3]. However, this is still far from achieving the global and Indian national target of elimination of parent-to-child transmission (EMTCT) and zero new paediatric HIV infections [1,4].

Given that 90% of HIV transmissions in children occur during pregnancy, labour, or breastfeeding; mothers can significantly reduce the risk of infecting their new-born to less than two percent by following the prevention of parent/(mother)-to-child transmission of HIV (PPTCT/PMTCT) program services [5]. Although the scope of the Indian PPTCT program is advanced and broad, it is estimated that only half of 27 million pregnant women are able to avail HIV testing services, and 84% of diagnosed HIV-positive mothers are able to receive antiretroviral prophylaxis [1,6,7].

**Figure 1:**
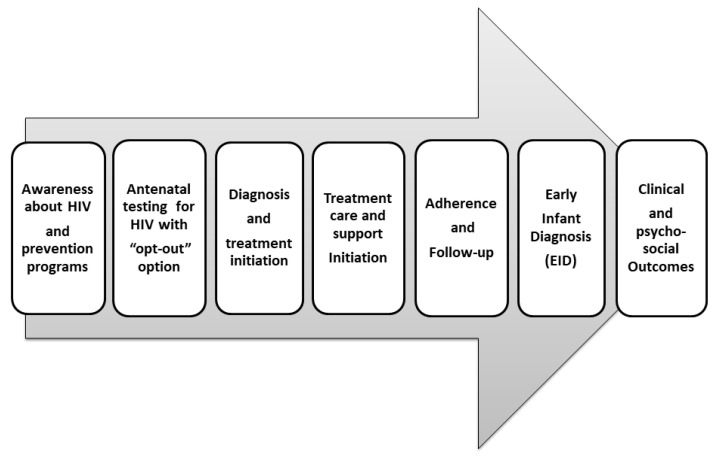
Prevention of Mother/Parent to Child Transmission (PMTCT/PPTCT) of HIV program cascade of care continuum.

The World Health Organization (WHO) recommends that women living with HIV navigate PMTCT services at definite time intervals from conception of the foetus to eighteen months after birth, as explained in Figure 1 [1,4,6]. Throughout this long process of care, multiple individual and environmental factors prevent pregnant woman from using HIV preventive services [5,6,7]. A systematic review on Indian PMTCT reports that most research focuses on quantitative summarizing of drop-out rates, while less attention is given to factors responsible for inadequate use of available healthcare [6,8]. Given the low numbers of pregnant women receiving HIV testing in India, and large number of gaps at each step of the care continuum in antenatal, intranatal, and postnatal phases, it is both urgent and important to understand barriers and facilitators of this PMTCT [9].

International qualitative research suggests that country, region and context-specific factors play a major role in deciding HIV prevention and care program adherence and outcomes [5,10,11]. It is also recognized that male partner/peer involvement is a salient interpersonal factor that influences maternal and paediatric HIV outcomes [10,12]; this may be particularly true in India, as men are the chief decision makers in the family.

This background on utility of PMTCT and its outcomes recognizes the need to explore barriers and solutions in individuals from diverse backgrounds with the help of refined methods [13]. The qualitative study reported herewith used the Socio-ecological Model (SEM) to explore facilitators and barriers that influence mothers’ decisions to initiate and adhere to the PMTCT cascade in western India. The SEM was used to explore the interface at which HIV-positive pregnant women interact with their peers, communities, and health system [5,11]. It suggests that women’s choices and circumstances leading to suboptimal PMTCT utilization are influenced by different levels of social organization embedded across five nested, hierarchal dimensions: individual, interpersonal, community, organizational, and policy [5,11]. We anticipate that findings from this study will inform urgently needed strategies/interventions that strive to achieve comprehensive adherence to the PMTCT program.

## 2. Methods

### 2.1. Study Design

According to the National AIDS Control Organization (NACO) guidelines, all pregnant women should be given the option of “provider-initiated” HIV testing and counselling as part of routine antenatal check-ups with the “opt-out” option (Figure 1). Those women who are diagnosed with HIV should be linked with PPTCT and anti-retroviral therapy (ART) centres for recommended HIV prevention, care, and support services [1]. This qualitative study explored disclosure, utilization experiences, and factors influencing uptake of the PMTCT program. Initial findings focused on HIV disclosure-related experiences and outcomes were published elsewhere [14]. In the present study, we present the factors influencing the utilization of PMTCT from the perspective of both members of the parental dyad.

### 2.2. Study Setting

The present study was conducted at a western Indian tertiary care institute and the attached ART centre offering NACO recommended PMTCT program services to all antenatal women.

### 2.3. Participants and Recruitment

Mothers and/or fathers of HIV-exposed infants were recruited to participate in the study. Mothers living with HIV were eligible to participate if they: (1) were 18 years or older; (2) provided informed consent; (3) had utilized any component of the PPMCT program during pregnancy, labour, or post-delivery; and (4) were able to speak local languages (i.e., Hindi or Gujarati). Fathers were eligible to participate if they: (1) were a partner of a mother living with HIV who had or were using PPMCT services; (2) were 18 years or older; (3) provided informed consent; and (4) were able to speak local languages.

At the ART centre, one investigator screened men, women, and couples for history of having an HIV-exposed child younger than two years of age. With the help of a standardized participant information sheet (PIS), another investigator explained the study to potential study participants. Interested individuals were assessed for eligibility and invited to provide written informed consent.

### 2.4. Ethical Clearance

An Ethics Committee and Institutional Review Board approved all procedures and materials prior to participant enrolment and data collection. The standards for reporting qualitative research (SRQR) were followed to prepare the present research article [15].

### 2.5. Study Sample Size and Sampling

The maximum variation purposive sampling and saturation theory tenets were followed to ensure exploration of responses from study cases having different levels of engagement and adherence in the PPTCT program [16]. Sixteen mothers and fifteen fathers, including six couples, were enrolled in the study (Table 1).

### 2.6. In-Depth Interview Guide for Data Collection

**Figure 2:**
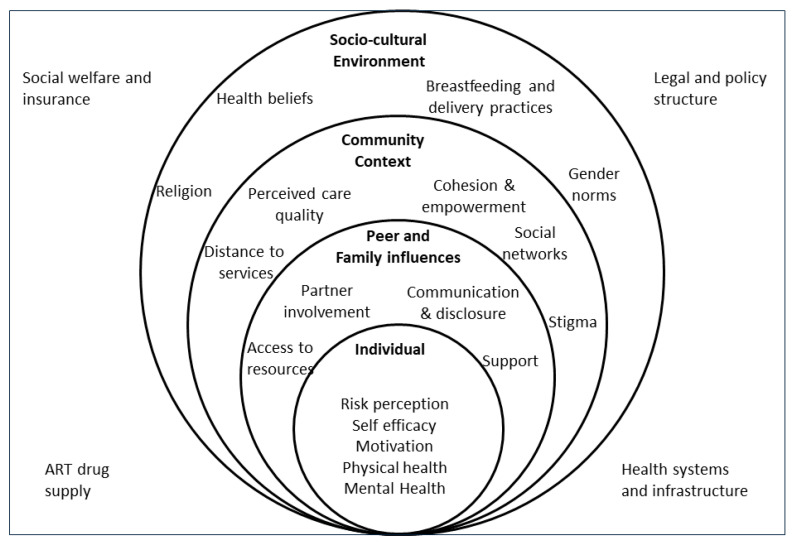
Social ecological framework for determinants of uptake, adherence and retention in Prevention of Mother to Child Transmission (PMTCT) program (adapted from Busza et al., 2012).

Study investigators conducted a literature review and engaged in discussions to develop and finalize a codebook, which was later used to frame the in-depth interview guide. The SEM main themes/dimensions, namely, individual, interpersonal, community, organizational, and policy (Figure 2), were categorized into “a priori” codes and sub-codes to prepare the code-book [11]. The interview guide was translated and piloted with local languages before administration. After the interview, a standardized questionnaire was verbally delivered to assess place of delivery and whether anti-retroviral prophylaxis was given during the last delivery for prevention of vertical transmission of HIV.

### 2.7. Data Processing and Analysis

The in-depth interviews were audio-recorded, transcribed verbatim, and translated into English by one author (AM). Two investigators read transcripts independently to familiarize themselves with the data, identify emerging codes, and code the text using the direct content analysis method [17]. Coding reports were discussed among all investigators to reach consensus in a series of meetings. Atlas Ti and Microsoft Excel were used to manage qualitative and demographic data analysis, respectively.

## 3. Results

### 3.1. Participant Characteristics

The majority (97%) of participants completed their education to less than 12th standard. Seventy five percent (*n* = 23) of pregnant mothers had their delivery at public institutions, twenty percent (*n* = 6) delivered at private hospitals, and the rest (*n* = 2) had a home delivery. More mothers had vaginal (*n* = 16) delivery than caesarean delivery (*n* = 15). Six women did not use antiretroviral prophylaxis immediately after birth of their baby (Table 1). Two fathers reported that their newborn died after their wife had a home delivery. Two mothers reported that their baby had become HIV-positive through vertical transmission. Two different women reported that their present baby was HIV-negative, but their previous baby had died from HIV-related infections.

### 3.2. Qualitative Analysis

**Figure 3:**
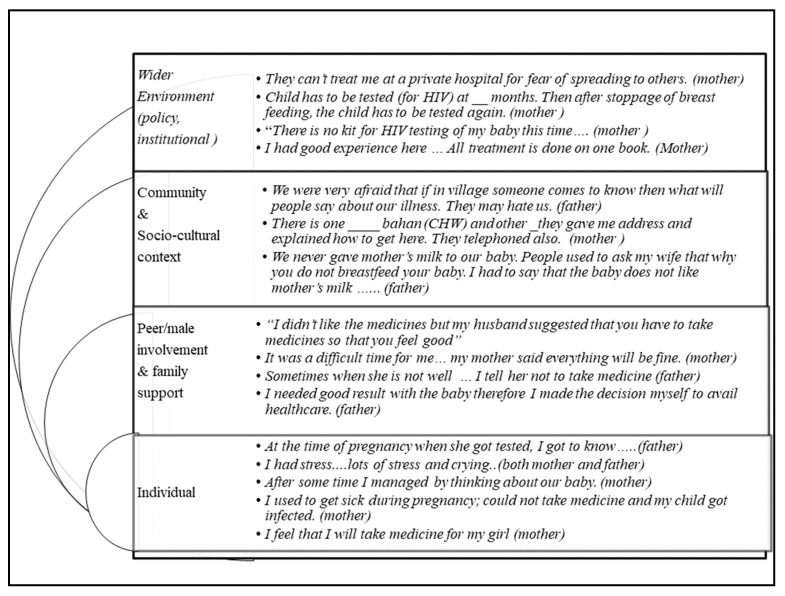
Barriers and Facilitators of Engagement in Prevention of Mother/Parent to Child Transmission (PMTCT/PPTCT) of HIV Services.

Thirty-one interviews (mean length: 25 min) were conducted until achieving saturation of “a priori” and emerging codes based on SEM. The main codes (Figure 2) and quotes which illustrate these codes are provided in the text below, Table 2, and Figure 3.

#### 3.2.1. Individual

Most study participants (*n* = 23) accessed HIV testing during routine pregnancy check-ups; however, sometimes women (*n* = 5) sought healthcare later in pregnancy (Table 1). A father reported that his pregnant wife learned about her positive HIV status only after being admitted to a hospital as a result of experiencing poor physical health, *“When my wife was five or six months pregnant, she was ill and getting weaker day by day. I admitted her at hospital for treatment. She was reported with HIV”.* After being diagnosed with HIV, pregnant women felt distressed, but the “hope of getting a healthy new baby” encouraged them to pursue PPTCT services. Some mothers mentioned that “sickness” and side effects prevented them from taking medication as prescribed by the doctor (Table 2 and Figure 3).

#### 3.2.2. Male Involvement

Most husbands recognized the importance of providing support when their wife was undergoing PPTCT recommendations, *“I gave courage to my wife… let happen which ever happened”.* Conversely, few fathers did not help mothers to seek PPTCT services, *“My husband never came with me whenever I came here. Now he is off (dead) and nothing.”* It is also worth mentioning that 84% of male partners in this study were living with HIV.

#### 3.2.3. Support from Peers and Family

Maternal relatives and a few friends supported parents living with HIV. A woman said, *“Everyone at home said that you should keep the child and whatever will happen will be seen later on”.* One father explained that he received help from friends, *“One friend does not think it is bad that I have HIV. If I am sick and have to stay at hospital; he watches my child.*” On the other hand, sometimes disclosing HIV status led to poor peer/family support. One father described a bad experience after revealing his positive HIV status to family members, *“…everyone, especially papa, used to be very angry… he said you will give it to others also”* (Figure 3). HIV disclosure seemed to be the most salient factor that influenced the level of peer and family support mothers received. Several fathers and mothers shared, “*If people have information about HIV, then I will tell them but, if the opposite person did not have such information, then they will think it is wrong and bad…*” The disclosure-related experiences and outcomes were published in a separate paper [14].

#### 3.2.4. Community Context

Parents preferred living in cities because they perceived more HIV stigma in rural areas. Their neighbours and acquaintances asked unwanted questions about their monthly full day absence from home, “*I have to come here to the hospital to take medicine and for follow-ups of my wife and child. I feel a lot of tension in coming here. People think that where these people go*”. Moreover, mothers shared their experiences with some healthcare providers who reinforced existing discrimination and stigma by refusing to provide them HIV care (Figure 3).

At the community level, ORW (outreach workers who are locally called “ben” meaning “sister”) encouraged engagement in the PPTCT care continuum by educating and helping parents navigate healthcare services. However, parents faced difficulties in adhering to treatment as a result of having to spend money and time on transportation or missing wages. One father said that “*I face difficulty in coming here from a distance. My village is far from here. If I come here, Rs____ (money) are needed for transportation.*” (Table 2).

#### 3.2.5. Sociocultural Factors

In India, breastfeeding is universally practiced, although PPTCT recommendations discourage mixed feeding (breastfeeding along with occasional liquids including milk other than breast milk) [1]. Parents who decided not to follow the usual cultural infant feeding practices faced disapproval from relatives and neighbours (Figure 3).

#### 3.2.6. Wider Environment Factors

At the institutional level, facilitators of engagement in PPTCT were good quality care at negligible prices, support from paramedical and medical staff, availability of multi-specialists at one location, and priority provided to mothers with young children. However, many improvements can be made because some parents reported unavailability of resources and improper care at a healthcare centre (Figure 3). One father who had stopped taking medication for himself while his wife continued to use PMTCT said, *“I did not admit my child in Civil* (government hospital). *There they make you run a lot. My wife has to run alone when I am at work.”* Several parents reported that private practitioners denied them care after mothers or their children were diagnosed with HIV: “*The child died because nobody admitted in private hospital and I did not know that we could admit him in Civil hospital*”.

The PPTCT program policy requesting the use of the early infant diagnosis tool (EID) to screen new-born’s for HIV at pre-fixed intervals motivated adherence. Regular growth monitoring and check-ups also served as important reminders for PMTCT check-ups (Table 2 and Figure 3).

## 4. Discussion

This is the first study in India, to the best of our knowledge, to apply the SEM to code and analyse in-depth interview data about a PMTCT program from both members of the parental dyad. The results from the present study indicate that adherence to PMTCT care continuum involves various personal, societal, and wider environmental factors (Table 2 and Figure 2 and Figure 3). Furthermore, this study provided notable insights into adherence to PMTCT (Table 2 and Figure 2 and Figure 3). Some (*n* = 5) parents did not obtain HIV testing during early pregnancy and some participants (*n* = 5 fathers) reported that their child was not provided post-exposure prophylaxis after birth. The majority (*n* = 26) of mothers learned about their HIV status during routine antenatal testing or when they developed symptoms of HIV-related illnesses, which provided a shorter time-period for post-test counselling and fewer opportunities for coping with HIV status and implementation of the PMTCT services [9,18]. On the other hand a few parents reported that their last HIV-exposed child survived and remained negative when the mother followed the prevention program [7].

In this study, none of the pregnant mothers decided on their own to avail healthcare services and needed their husbands or family members to make the decision for them. Close relatives or/and friends encouraged parents to use ‘medicine’ and adhere to ‘doctor’s advice’ to normalize their situation of living with HIV. Previous systematic reviews [6,11,12] and an Indian private sector study [19] also found that women rely on men’s approval and motivation when deciding to avail health services to prevent paediatric HIV infection. Studies across the globe and WHO guidelines have recognized the importance of family counselling in the elimination of newborn HIV infections [13].

At the socio-cultural and community level, community health/outreach workers (CHWs)/ORWs facilitated linking of parents to the PMTCT program, while lack of financial resources, transportation, and knowledge about HIV limited parents’ access to the PMTCT cascade. Similar to other studies of the PMTCT program [6,11], HIV stigma was a major barrier to the HIV care continuum in the current study. This research further clarifies that knowledge about prevention and care programs may reduce HIV-associated stigma in friends and family. On the other hand, stigmatizing attitudes in the community and among healthcare workers may purport “internalized stigma” [20] also known as self-stigma (acceptance of stigma as valid).

At the wider environment level, the NACO policy of implementing the early infant diagnosis (EID) tool for an HIV-exposed baby, provision of free undisturbed drug supply, separate clinics for mother–baby, counselling, growth monitoring and regular check-ups in public institutions were important to help parents to obtain care across the PMTCT care continuum. Positive attitudes of healthcare providers motivated adherence to PMTCT. A novel finding in this study indicated that denial of healthcare by private hospitals or care providers prevented timely uptake of HIV care, which might impede prevention of transmission of HIV to the newborn [8,20]. Considering that seventy percent of Indian population prefers to avail medical care from the private sector [18], lack of pre-test/post-test counselling and linkage to available antiretroviral therapy by healthcare providers in private hospitals and clinics presented a major setback to early initiation of the PMTCT services. This barrier, further enforced by peer, family and society’s lack of knowledge about HIV care, limited pregnant women and mother’s adherence to the stepwise PMTCT measures, at the intrapersonal level [19]. Research from other regions of the Indian sub-continent confirms that mainstreaming and capacity building of the private health sector is needed [18].

### 4.1. Limitations

These qualitative findings follow rigorous study methods and are inclusive of fathers/male partners, but similar to all qualitative studies, may not transfer to parents outside the study setting, especially for those who are lost to follow-up or who withdraw from care. We recorded responses from both members of parental dyads, which was an overall strength of the present study, but given that all participants were recruited from a PMTCT program, this could have amplified husbands’/partners’ support towards HIV-positive women and PMTCT/PPTCT in India. Women were interviewed a few months after childbirth, which could have led to recall bias of more negative and acute problems in early pregnancy.

### 4.2. Recommendations for Practice and Policy

The present results have important implications for PPTCT programs in India and potentially other similar locations. First, given that other people may significantly influence women’s health care decisions, there is an urgent need for strategies to reduce “internalized stigma” from family and the private health sector to ensure timely referral to PMTCT services. Second, although the reported quality of care at the public ART centre may be good overall, some improvements can be made with availability of resources and skills. Third, when other people had negative attitudes and misconceptions about HIV/AIDS, they were less likely to help mothers avail healthcare from prevention programs. Locally relevant strategies to sensitize and educate fathers, peers, family, and private healthcare providers about the benefits and availability of PPTCT care can ensure reduction of stigma, comprehensive adherence, and prevention of newborn HIV infections.

## 5. Conclusions

The present study suggests that interpersonal support from partners, peers, and family can improve mothers’ adherence to PMTCT services. Future intervention strategies should seek to develop and improve pregnant women’s support systems by engaging empathetic husbands/partners, family members, social/community workers, and healthcare providers. Strategies addressing the private medical sector, and awareness about HIV prevention may enable optimal PPTCT utilization.

## Figures and Tables

**Table 1:** Socio-economic and related characteristics of PMTCT beneficiaries.

Characteristic	Mother (*n* = 16)	Mother %	Father (*n* = 15)	Father %	Total (*n* = 31)	Total (%)
Point of HIV Testing of Pregnant Mother						
Routine ANC testing (mother)	14	87.5	12	80	26	83.9
Both parents tested together	5	31.6	5	33.3	10	32.3
Mother tested later due to sickness	2	12.5	3	20.0	5	16.2
Husband positive before mother	7	43.8	6	40.0	13	41.9
Education						
Inability to read and write or no formal education	4	25.0	0	0	4	12.9
Primary (able to read and write but completed less than 7th standard)	9	56.3	7	46.7	16	51.6
Secondary (completed 7th standard but less than 12th standard)	2	12.5	8	53.3	10	32.3
Higher-Secondary (completed 12th standard) or/and above	1	6.2	0	0	1	3.2
Place of Delivery						
Government hospital	13	81.3	10	66.7	23	74.2
Private hospital	3	18.7	3	20.0	6	19.4
Home	0	0	2	13.3	2	6.4
Type of Delivery						
Caesarean section	9	56.3	6	40.0	15	48.4
Vaginal	7	43.7	9	60.0	16	51.6
Nevirapine (NVP) Prophylaxis Administered to New-born						
Yes	15	93.8	10	66.7	25	80.6
No	1	6.2	5	33.3	6	19.4

**Table 2:** SEM facilitators and barriers in PPMCT.

Themes	Facilitators	Barriers
Intrapersonal and Interpersonal Socio-economic Factors		
Individual	Routine antenatal HIV testing Hope for a new baby Knowledge about medical care available	Feeling of anxiety Poor awareness about PMTCT Feeling betrayed by partner (husband positive before wife)
		
Interpersonal/Male involvement	HIV-positive status of men Men’s awareness about PMTCT and ART	Negative father and positive mother Mother tested positive during routine ANC testing (before father)
		
Peer and family influences	Educational status of family members Awareness about available health and support facilities	Fear of disclosure and repercussions Poor awareness about HIV prevention, care and support program
		
Community and socio-cultural context	Social network of positive people Outreach (Community) workers Public health services Perceived good quality care	HIV-associated stigma Distance to travel Village/rural settings Expectations of breastfeeding baby Private healthcare providers enforced internalised (self) stigma
		
Wider Environment Factors		
Health system and infrastructure	Caring attitude and information provided by healthcare provider Linking of PPMCT and ART centre Counselling of partners and family members by trained staff	Separate mother and child health-care departments Private hospitals testing and treatment policies Referral to other setups
		
Health Policies	Early infant diagnosis tool (EID) Growth monitoring Periodic check-ups of mother and baby Antiretroviral prophylaxis for HIV exposed baby	Unavailability of resources in public healthcare sector Non-uniform and unregulated implementation of PPTCT program by private medical sector

## Data Availability

The data supporting reported results are narratives of parents and can be obtained from the first author via email or contact information. The public archiving of datasets has to follow institutional ethical approval committee rules.
